# 

**Published:** 1857-09

**Authors:** 


### CONTENTS.

### *ORIGINAL COMMUNICATIONS.*

PAGE.

ARTICLE I.—Paris Correspondence. By THOMAS A. MEANS, M. D., Oxford, Geo.—now in France,........................................................ 1

ARTICLE II.—Something "New under the Sun!" Negro Testimony *vs.* The Science of Medicine, &c. By J. BORING, M. D............................ 14

ARTICLE III.—A Case of Menorrhagia and Treatment. By E. C. MOYER, M. D., of Talbotton, Geo............................................ 16

ARTICLE IV.—Chloroform. By J. S. WEATHERLY, M. D., of Montgomery, Alabama,................................................................ 21

ARTICLE V.—On the use of Belladonna, in arresting the secretion of milk. By J. H. Connahy, M. D., Barnesville, Ga., April 10, 1857.................. 24

### *SELECTIONS.*

Sudden Deaths in the Puerperal state, &c.,.......................... 25

The Medical Treatment of Insanity,.................................... 28

Review of Nature and Art in the cure of Disease,...................... 33

The "Hotel Endemic" at Washington,.................................... 44

Pork and Potatoes—their Influence on the Bodies and Minds of our People.... 49

### *EDITORIAL AND MISCELLANEOUS.*

The Third Volume,........................................................ 54

American Medical Association and Medical Education,..................... 55

The late meeting of the American Medical Association at Nashville......... 57

Close of the Session,.................................................... 59

Fiske Medical Prize,..................................................... 59

Gelseminum Sempervirens in Gonorrhœa,..................................... 60

New York State Lunatic Asylum,............................................ 60

Consolidation of Medical Schools,......................................... 60

Ergot in Menorrhagia,..................................................... 60

Preservation of Vaccine Virus,............................................ 61

Singular case of Lactation,............................................... 61

Iodide of Calomel,........................................................ 62

Dysentery,................................................................ 62

Interesting to Physicians,................................................ 62

Electricity in the Suppression of the Lacteal Secretion................... 63

Ergotine in Epidemic Diarrhœa,............................................ 63

Arsenic and Stillingia in Diseases of the Skin............................ 63

Meteorological Table,..................................................... 64

